# Peptide Inhibition of Topoisomerase IB from *Plasmodium falciparum*

**DOI:** 10.4061/2011/854626

**Published:** 2011-05-04

**Authors:** Amit Roy, Ilda D'Annessa, Christine J. F. Nielsen, David Tordrup, Rune R. Laursen, Birgitta Ruth Knudsen, Alessandro Desideri, Felicie Faucon Andersen

**Affiliations:** ^1^Department of Molecular Biology and Interdisciplinary Nanoscience Center (iNANO), Aarhus University, C.F. Møllers Allé 3, Building 1130, 8000 Aarhus C, Denmark; ^2^Department of Biology and Center of Biostatistics and Bioinformatics, University of Rome “Tor Vergata”, Via della Ricerca Scientifica 1, 00133 Rome, Italy; ^3^Interuniversity Consortium, National Institute Biostructure and Biosystems (I.N.B.B.), 00136 Rome, Italy

## Abstract

Control of diseases inflicted by protozoan parasites such as *Leishmania, Trypanosoma*, and *Plasmodium*, which pose a serious threat to human health worldwide, depends on a rather small number of antiparasite drugs, of which many are toxic and/or inefficient. Moreover, the increasing occurrence of drug-resistant parasites emphasizes the need for new and effective antiprotozoan drugs. In the current study, we describe a synthetic peptide, WRWYCRCK, with inhibitory effect on the essential enzyme topoisomerase I from the malaria-causing parasite *Plasmodium falciparum.* The peptide inhibits specifically the transition from noncovalent to covalent DNA binding of *P. falciparum* topoisomerase I, while it does not affect the ligation step of catalysis. A mechanistic explanation for this inhibition is provided by molecular docking analyses. Taken together the presented results suggest that synthetic peptides may represent a new class of potential antiprotozoan drugs.

## 1. Introduction

Protozoan parasites, such as *Leishmania*, *Trypanosoma*, and *Plasmodium *species are the cause of a large array of diseases hampering the lives of people all over the world [1]. Control of such diseases depends on a rather small number of prophylactic or therapeutic antiparasite drugs, many of which are highly toxic and/or inefficient [2–5]. In addition, an increasing number of parasites develop resistance towards several of the frontline drugs [6–9]. This has created an urgent need for novel compounds to prevent and cure diseases caused by protozoan parasites. Species-specific inhibition of parasitic enzymes has been suggested as one promising approach in the development of new therapeutics [10]. One family of enzymes that have attracted considerable interest as potential targets for antiparasitic therapeutics are the DNA topoisomerases (Topos) [1, 11] of which the human counterparts are well-known targets in anticancer treatment [12]. 

DNA Topos are ubiquitous enzymes needed to overcome the topological stress arising in DNA during replication, transcription, recombination, and repair [13]. This is achieved by the enzymes introducing transient breaks in the DNA in a reaction that restores the energy of the broken phosphodiester bond in a covalent phosphotyrosyl cleavage intermediate. Based on their mechanism of action Topos are divided into two main classes [13, 14]. The type I Topos are with few exceptions monomers and relax DNA by breaking only one strand of the double helix, while type II Topos are mainly homodimers or heterotetramers and break both strands of the DNA simultaneously. Type I Topos are further classified into two structurally unrelated families denoted the type IA and type IB Topos, defined by the polarity of their strand cleavage. The type IA Topos are prevalent in prokaryotic species and create a 5′-phoshotyrosyl linkage and a free 3′-OH DNA end during cleavage. Type IB Topos are mainly found in eukaryotic species and generate a 3′-phosphotyrosyl linkage and a free 5′-OH DNA end during cleavage. The class of type II Topos are subdivided into the type IIA and type IIB families, of which all members are structurally related and characterized by the formation of a 5′-phoshotyrosyl linkage and a free 3′-OH DNA end during cleavage. The type IIA Topos are found both in eukaryotic and prokaryotic species. Typically the eukaryotic members of this group are homo-dimers while the prokaryotic enzymes are heterotetramers [14]. The type IIB group encompasses TopoVI of extreme thermophilic archaebacteria [15]. 

Besides their important biological functions, DNA Topos from the various groups are well-known targets of both antibacterial and anticancer therapeutic agents. Hence, bacterial type IIA Topos, such as DNA gyrase and TopoIV, are targets of clinically important antibiotics active against a wide spectrum of bacterial pathogens [16, 17]. Human type IB and IIA Topos are targets of several anticancer compounds, exemplified by camptothecin and etoposide, respectively, of which synthetic derivatives are routinely used in systemic treatment of different cancer types [12, 18]. Of relevance for the treatment of protozoan-caused infectious diseases, structural and/or subtle mechanistic differences between protozoan and host Topos can be exploited for the rational design of novel therapeutic compounds. Indeed, the unusual heterodimeric TopoIB of kinetoplastid parasites, such as *Leishmania donovani *gives hope for the development of drugs targeting parasite TopoIB without interfering with the function of the monomeric TopoIB in the human host [11, 19, 20]. As another example, the apicomplexan parasite *Plasmodium falciparum* contains apicoplast DNA, which requires bacterial-type DNA gyrases (type IIA Topo) for replication, thus providing a unique drug target absent in the host [21, 22]. In addition, the high expression rate of TopoIB and TopoIIA in rapidly growing parasites, compared to the expression levels of these enzymes in the host, may be exploited for the development of Topo-targeting protocols that specifically kill the parasites. 

Synthetic peptides have been prophesied to be the ideal inhibitors of enzyme activity either alone or in combination with small-molecule drugs [10, 23]. However, high synthesis costs and great challenges regarding delivery, intracellular targeting and clearance half-life of peptides have until recently hampered the interest of most pharmaceutical companies in developing peptide-based drugs. New efficient synthesis strategies and low monomer prices have led to a renewed interest in therapeutic peptides. Indeed, compared to small-molecule drugs, which are currently dominating the pharmaceutical market, peptide-based therapeutics offer several advantages, such as high specificity, lower accumulation in tissues, lower toxicity, and biological diversity [24–27]. 

The potential for synthetic peptides as efficient species-specific inhibitors even of discrete steps of Topo catalysis is highlighted in several studies by Nagaraja and co-workers describing the identification and characterization of species-specific antibodies with inhibitory activities against particular steps of *Mycobacteria* DNA gyrase or TopoI catalysis [28–30]. Peptides with similar inhibitory activities and potential in future antituberculosis treatment [29] are likely to be derived from such antibodies [31–34]. Relevant for the potential development of peptide-based drugs targeting eukaryotic Topos, almost a decade ago, Segall and co-workers identified a series of hexapeptides inhibiting various catalytic steps of the tyrosine recombinases (bacteriophage *λ*-Int and Cre) [35, 36]. Tyrosine recombinases share so important structural and mechanistic features with the type IB Topos that they can be considered a subbranch of the type IB Topo family [37, 38]. It was therefore not surprising, that several of the hexapeptides selected on basis of *λ*-Int inhibition also inhibited DNA relaxation by the type IB Topo of *Vaccinia virus* (vvTopoI), although less potently [39]. A rescreening of the peptide combinatorial library (used for selection of the above-mentioned peptides) specifically against vvTopoI resulted in the identification of three new peptides, WYCRCK, KCCRCK, and WRWYCRCK with high activity against this enzyme. Of these, WRWYCRCK was the most potent inhibitor of the type IB Topos tested. This peptide inhibited vvTopoI with an IC_50_ value of 0.1–0.25 *μ*M and *λ*-Int with an IC_50_ value of 0.015 *μ*M, while the structurally unrelated type IA Topo, *E. coli* TopoI was inhibited only to a limited extent (IC_50_ value of 5.5 *μ*M) [40]. Using these peptides as a starting point, it may in longer terms be possible to develop peptide-based TopoI targeting inhibitors with therapeutic activity against protozoan pathogens. 

As an initial investigation of this possibility, we address, in the present study, the effect of the peptides WYCRCK, KCCRCK, and WRWYCRCK on the activity of the recently cloned and purified recombinant TopoI (pfTopoI) from the malaria-causing parasite *Plasmodium falciparum*. We find that WRWYCRCK inhibits DNA relaxation and cleavage by pfTopoI whereas neither WYCRCK nor KCCRCK have any effect on pfTopoI activity. Molecular docking of the three peptides in the noncovalent pfTopoI-DNA complex shows WRWYCRCK to be located in the minor groove of the DNA in proximity of the enzyme active site, while WYCRCK and KCCRCK are positioned far from the enzyme active site.

## 2. Methods

### 2.1. Expression and Purification of pfTopoI

The plasmid, pPFT100 (the cloning of pfTopoI is to be published elsewhere), containing the pfTopoI gene (PlasmoDB accession number PFE0520c) [41] (codon optimized for expression in *S. cerevisiae* (GENEART, Germany)), was transformed into the yeast *S. cerevisiae *top1Δ strain RS190 (a kind gift from R. Sternglanz, State University of New York, Stony Brook, NY, USA) according to standard procedures, and pfTopoI enzyme was expressed and purified as previously described for human topoisomerase I (hTopoI) [42]. hTopoI was expressed and purified as previously described [42].

### 2.2. Unit Definition

1 U is the amount of enzyme needed to fully relax 200 fmol of negatively supercoiled pBR322 plasmid DNA at 37°C in 30 min in 10 mM Tris (pH 7.5), 1 mM EDTA, 150 mM NaCl, 5 mM MgCl_2_ and 5 mM CaCl_2_.

### 2.3. Synthetic Peptides

WYCRCK, KCCRCK, and WRWYCRCK were purchased from GenScript USA Inc., USA. The lyophilized peptides were dissolved in H_2_O.

### 2.4. Relaxation Assays

DNA relaxation reactions included 1 U pfTopoI in the absence or presence of peptide (WYCRCK, KCCRCK, or WRWYCRCK) at the following concentrations: 1.3 *μ*M, 2.5 *μ*M, 5 *μ*M, 7.5 *μ*M, 12.5 *μ*M, 25 *μ*M, or 50 *μ*M and 200 fmol negatively supercoiled pBR322 plasmid in 20 *μ*L of 10 mM Tris (pH 7.5), 1 mM EDTA, 150 mM NaCl, 5 mM MgCl_2_ and 5 mM CaCl_2_. The plasmid was preincubated with the peptide for 5 min at 37°C prior to addition of enzyme. Reactions were incubated at 37°C for 30 min before being stopped by addition of 0.2% (w/v) SDS and proteolytically digested with 0.5 *μ*g/mL proteinase K for another 30 min at 37°C. Samples were subjected to gel electrophoresis on 1% agarose gels in TBE buffer, and DNA bands were stained with ethidium bromide and visualized by illumination with UV light.

### 2.5. Synthetic DNA Substrates

Oligonucleotides for assembly of DNA suicide cleavage substrates and DNA ligation substrates were purchased from DNA Technology, Denmark and purified by denaturing polyacrylamide gel electrophoresis. The sequences of the substrates are as follows: OL19: 5′-GCC TGC AGG TCG ACT CTA GAG GAT CTA AAA GAC TTA GA-3′, OL27: 5′-AAA AAT TTT TCT AAG TCT TTT AGA TCC TCT AGA GTC GAC CTG CAG GC-3′, and OL36: 5′-AGA AAA ATT TTT-3′. The oligonucleotide representing the scissile strand (OL19) was 5′-radiolabeled by T4 polynucleotide kinase (New England Biolabs, USA) using [*γ*-^32^P] ATP as the phosphoryl donor. To prevent ligation of the 5′-OH from the bottom strand (OL27), these ends were 5′-phosphorylated with unlabeled ATP. The oligonucleotides were annealed pairwise with a 2-fold molar excess of the bottom strand over scissile strand as previously described [43].

### 2.6. Cleavage/Ligation Assays

The cleavage reactions were carried out in 20 *μ*L reaction volumes by incubating 20 nM of the duplex OL19/OL27 with 500 fmol of pfTopoI or hTopoI enzyme at 37°C, in 20 mM Tris (pH 7.5), 10 mM MgCl_2_, and 10 mM CaCl_2._ The DNA substrate was preincubated with peptide WRWYCRCK at concentrations varying from 0 to 75 *μ*M for 5 min at 37°C prior to addition of enzyme. After 30 minutes of incubation, the reactions were stopped with 0.1% (w/v) SDS. For the ligation reactions, 20 nM OL19/OL27 was incubated with 500 fmol of pfTopoI for 30 min at 37°C in 10 mM Tris (pH 7.5), and 5 mM MgCl_2_, 5 mM CaCl_2_. After preincubation of the cleavage samples with the peptide at concentrations varying from 0 to 12.5 *μ*M for 5 min at 37°C, ligation was initiated by the addition of a 200-fold molar excess of oligonucleotide OL36 over the duplex OL19/OL27. Samples were incubated at 37°C for 60 min, and reactions were stopped with 0.1% (w/v) SDS. Cleavage and ligation samples were precipitated with ethanol, resuspended in 10 *μ*L of 1 mg/mL trypsin, and incubated at 37°C for 30 min. Reaction products were analyzed by gel electrophoresis on 12% denaturing polyacrylamide gels, and radioactive bands were visualized by Phosphorimaging.

### 2.7. Quantification

Densitometric quantification of gel bands was performed using Quantity One v4.6.3 software (Bio-Rad, USA). The relative cleavage was calculated by the following equation: relative cleavage = (IC − BI)/(IC − BI + IS − BI), where IC denotes the intensity of the band(s) representing the cleavage product(s), IS denotes the intensity of the band representing the substrate, and BI denotes the background intensity.

### 2.8. Restriction Digestion of Plasmid in the Presence of Peptide

Restriction digests were performed in 20 *μ*L reaction volumes by incubating 3 *μ*g pUC19 plasmid with EcoRI or PvuII (both from New England Biolabs) in the reaction buffers provided by the manufacturer in the absence or presence of peptide WRWYCRCK (12.5 *μ*M, 25 *μ*M, or 50 *μ*M). The plasmid was preincubated with the peptide for 5 min at 37°C prior to addition of enzyme. For both restriction endonucleases, the lowest amount of enzyme, able to fully digest the plasmid within the timeframe of the experiment, was used. Reactions were incubated at 37°C for 30 min before being stopped by addition of 0.2% (w/v) SDS and proteolytically digested with 0.5 *μ*g/mL proteinase K for another 30 min at 37°C. Samples were subjected to gel electrophoresis on 1% agarose gels in TBE buffer, and DNA bands were stained with ethidium bromide and visualized by illumination with UV light.

### 2.9. Docking Experiment

The three-dimensional structure for residues Pro140-Phe839 of pfTopoI was obtained through homology modeling using the SwissModel server [44] and the crystal structure of hTopoI (1K4T and 1A36 PDBs) as a template [45, 46]. The alignment was performed with the TCOFFEE server [47], using the sequences having the SwissProt code Q26024 and P11387 for the pfTopoI and hTopoI protein, respectively. The 22-base-pair DNA present in the noncovalent hTopo1-DNA complex crystal structure 1K4S [46] was fitted into the putative pfTopoI active site in the 3D protein model to obtain the pfTopoI-DNA noncovalent complex that was used for the docking experiment. The bases are numbered from 1 to 22 starting from the 5′ end of the scissile strand and from 23 to 44 starting from the 5′ end of the intact strand. The structure of the octapeptide WRWYCRCK and of the two hexapeptides WYCRCK and KCCRCK was designed with the Sybyl v. 6.0 program (TRIPOS, http://www.tripos.com/) creating a disulphide bond between the two Cys3 and Cys5 cysteines in all the peptides (this was done since the experimental data confirmed that disulfide bridging was necessary for the inhibitory effect of the peptide). The structure of the peptides was minimized in vacuum using the Powell algorithm [48] implemented in the Sybyl program and then simulated in a rectangular box filled with water molecules using the Gromacs 4.0 Package [49] for 2 ns in order to regularize the structure. 250 docking runs were performed using the Autodock 4.2 program [50] using the Lamarckian genetic algorithm [51]. The structures of the ligands (WRWYCRCK, WYCRCK, or KCCRCK) and the receptor (pfTopoI-DNA complex) were first prepared using the AutodockTools v. 1.5.2 suite [50], building a cubic box able to contain the cap and cat domains of the protein and the DNA bases. The contacts between the ligand and the receptor were identified using a cutoff of 3.5 Å applying a modified version of the g_mindist tool present in the Gromacs 3.3.3 package for Molecular Dynamics analysis [52]. The images were created with the program VMD [53].

## 3. Results

### 3.1. Inhibitory Potency of Peptides WYCRCK, KCCRCK, and WRWYCRCK in Relaxation by pfTopoI

**Figure 1:**
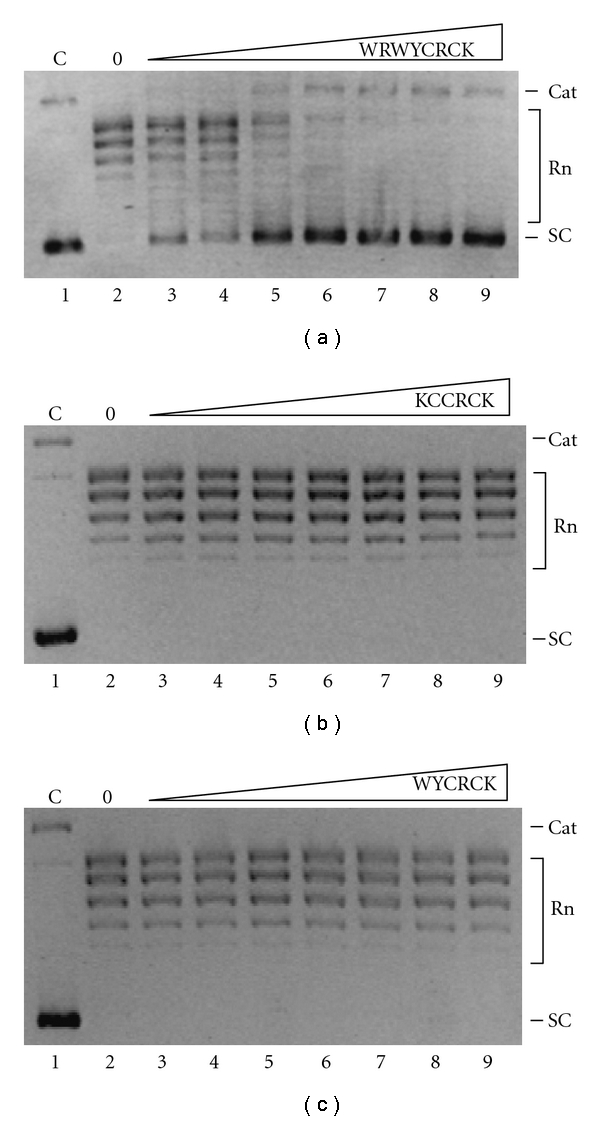
Effect of peptides on pfTopoI-mediated DNA relaxation. The effect of peptides on relaxation was assayed by incubating 200 fmol supercoiled plasmid with enzyme and peptide WRWYCRCK, KCCRCK, or WYCRCK at the following concentrations: 1.3 *μ*M, 2.5 *μ*M, 5 *μ*M, 7.5 *μ*M, 12.5 *μ*M, 25 *μ*M, or 50 *μ*M. (a) Representative gel picture showing the relaxation activity of pfTopoI in the presence of increasing concentrations of peptide WRWYCRCK. (b) Representative gel picture showing the relaxation activity of pfTopoI in the presence of increasing concentrations of peptide KCCRCK. (c) Representative gel picture showing the relaxation activity of pfTopoI in the presence of increasing concentrations of peptide WYCRCK. C: negative control lane without any enzyme added; 0: positive control lane with pfTopoI but no peptide added; SC: supercoiled plasmid; Rn: relaxed topoisomers; Cat: supercoiled catenated plasmid.

The inhibitory potency of the peptides WYCRCK, KCCRCK, and WRWYCRCK on pfTopoI activity was investigated in a standard plasmid relaxation assay. The assay was performed with the minimum amount of pfTopoI that sufficed to fully relax the plasmid DNA (i.e., convert fast-mobility supercoiled plasmid to slow-mobility relaxed plasmid forms) in the absence of added peptide within the timeframe (30 min) used in the experiment (data not shown). As evident from Figure 1, the peptide WRWYCRCK inhibited pfTopoI relaxation activity in a dose-dependent manner, with an IC_50_ of 2.5–5 *μ*M. The peptides WYCRCK, KCCRCK had no effect on the relaxation activity of pfTopoI, even at concentrations up to 50 *μ*M. Moreover, consistent with previous reports of inhibition of vvTopoI by WRWYCRCK, the peptide only retained its inhibitory effect in the absence of DTT (data not shown), suggesting that the active form of the peptide involves disulfide bridging. 

### 3.2. Inhibitory Potency of Peptide WRWYCRCK in Cleavage/Ligation by pfTopoI

**Figure 2:**
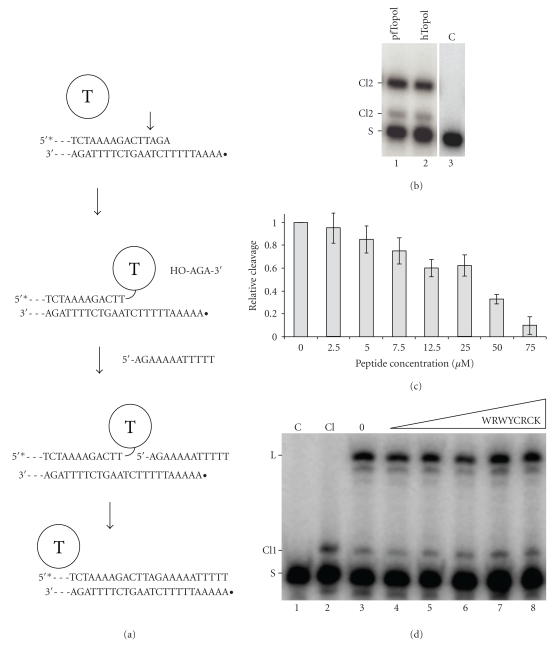
Effect of peptide WRWYCRCK on pfTopoI-mediated DNA cleavage. (a) Schematic depiction of the cleavage and religation reactions. The substrate (OL19/OL27) used for assaying cleavage allows covalent attachment of the enzyme to the 3′ end of the 5′-radiolabeled scissile strand (OL19) by cleaving off a trinucleotide. Ligation is prevented by diffusion of the trinucleotide. To initiate ligation, the ligator strand (OL36) is added to covalent cleavage complexes generated by incubating pfTopoI with radiolabeled OL19/OL27. (b) Gel picture showing the cleavage products obtained by incubating 5′-radiolabeled OL19/OL27 with pfTopoI (lane 1) or hTopoI (lane 2). (c) Graphical depiction of the cleavage activity of pfTopI plotted as a function of peptide WRWYCRCK concentration. The cleavage activity was calculated as described in Section 2. (d) Representative gel picture showing the ligation activity of pfTopoI in the presence of peptide WRWYCRCK at the following concentrations: 1.3 *μ*M, 2.5 *μ*M, 5 *μ*M, 7.5 *μ*M, or 12.5 *μ*M. T: topoisomerase I; asterisk: 5′-radiolabel with [*γ*-32P]; filled circle: 5′-cold phosphorylation; S: substrate; Cl1: cleavage product resulting from cleavage at the black arrow in the schematic depiction; Cl2: cleavage product resulting from cleavage two nucleotides upstream of the black arrow in the schematic depiction; C: negative control lanes without any enzyme added; Cl: cleavage control lane without ligator strand added; 0: positive control lane with pfTopoI but no peptide added.

DNA relaxation by type IB Topos involves two discrete transesterification reactions that is, a cleavage reaction, in which the active site tyrosine attacks the phosphodiester bond of the DNA backbone to generate a 3′-phosphotyrosyl cleavage intermediate and a free 5′-OH end, and a ligation reaction in which the 5′-OH acts as a nucleophile on the phosphotyrosyl bond to restore intact DNA. It was previously demonstrated that the inhibitory effect of the peptides WYCRCK, KCCRCK, and WRWYCRCK on DNA relaxation by vvTopoI and *λ*-Int could be ascribed to a specific inhibition of the cleavage and not the ligation step of catalysis [39, 40]. To address which steps of pfTopoI catalysis are affected by peptide WRWYCRCK, that inhibited relaxation by this enzyme we used a synthetic partially single-stranded suicide DNA substrate containing a preferred type IB TopoI cleavage sequence. This substrate, that was originally developed to investigate cleavage by hTopoI, acts as a mechanism-based inactivator of nuclear type IB Topos by allowing DNA cleavage, while the subsequent religation reaction is prevented due to diffusion of the generated 5′-OH end (see Figure 2(a)). Prevention of religation, however, is only conditional and this step of catalysis can be initiated by the addition of a surplus of a 5′-OH-containing ligator strand with a sequence matching the protruding noncleaved strand of the generated cleavage complexes (Figure 2(a)). 

First, the ability of pfTopoI with cleave the suicide DNA substrate was tested in comparison to cleavage by hTopoI. The two enzymes were incubated with substrate radiolabeled at the 5′-end of the cleaved strand (to allow visualization of the cleavage products), the products were ethanol precipitated, trypsinated, and separated on a denaturing polyacrylamide gel prior to visualization by PhosphorImaging. As evident from Figure 2(b), pfTopoI cleaved the substrate and gave rise to cleavage products (marked Cl1 and Cl2) with approximately the same gel electrophoretic mobilities as those of cleavage products generated by hTopoI (compare lanes 1 and 2). These products were retained in the slot of the gel if trypsin digestion was omitted (data not shown), confirming their identity as covalent pfTopoI-DNA or hTopoI-DNA complexes. As previously reported in [54], even after trypsin digestion, the cleavage products of both pfTopoI and hTopoI were retarded in the gel due to the covalent attachment of short protease-resistant peptides to the radiolabeled strand of the DNA substrate. For hTopoI, the major cleavage product Cl1 was previously demonstrated to result from cleavage at the preferred site (indicated by an arrow in Figure 2(a)), while the minor Cl2 product arises from cleavage two nucleotides upstream to the cleavage site [54]. The gel electrophoretic mobility of cleavage products generated by pfTopoI suggests that this enzyme cleaves the utilized substrate at the same positions as does hTopoI.

To test the effect of peptide WRWYCRCK on pfTopoI mediated cleavage, increasing concentrations of the peptide were incubated with the above-described suicide DNA substrate prior to addition of pfTopoI. The reactions were performed essentially as described above and the percentage of substrate converted to cleavage product shown as a function of peptide concentration (Figure 2(c)). As previously reported for vvTopoI and *λ*-Int, peptide WRWYCRCK inhibited DNA cleavage by pfTopoI in a dose-dependent manner, although the observed cleavage inhibition was less potent than that observed for DNA relaxation. 

Using the suicide substrate system, the effect of peptide WRWYCRCK on pfTopoI-mediated religation was investigated. In this experiment, preformed cleavage complexes were incubated with increasing concentrations of WRWYCRCK prior to addition of the ligator strand shown in Figure 2(a). Consistent with previous results obtained for vvTopoI and *λ*-Int the peptide did not affect ligation by pfTopoI (Figure 2(d)).

### 3.3. Peptide Specificity

**Figure 3:**
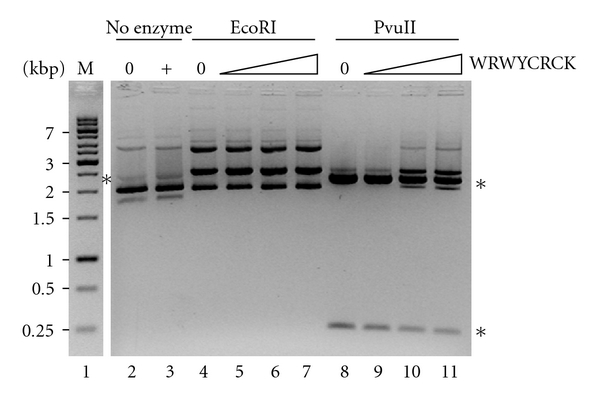
Effect of peptide WRWYCRCK on restriction digestion by restriction endonucleases. Representative gel picture showing the result of incubating pUC19 plasmid with EcoRI (lanes 4–7) or PvuII (lanes 8–11) in the presence of peptide WRWYCRCK at the following concentration: 12.5 *μ*M, 25 *μ*M, or 50 *μ*M. The sizes, kbp, of the DNA marker (lane 1, labeled M) are shown to the left of the gel picture. 0: control lanes with no peptide added; +: control lane with 50 *μ*M peptide added; asterisks indicate the gel electrophoretic mobility of the digested plasmid, for EcoRI, 2.7 kbp, and for PvuII, 0.3 kbp and 2.4 kbp.

The three peptides tested for activity against pfTopoI in the present study were previously demonstrated to inhibit vvTopoI and *λ*-Int activity with IC_50_'s of 0.015–2.3 *μ*M, while more unrelated enzyme activities such as *E. coli *type IA Topo and restriction endonucleases were hardly affected by any of the peptides. Although far from being species-specific, the peptide inhibitors appear rather sensitive to even subtle structural differences between the different target enzymes. This is evident from the different inhibition pattern of pfTopoI observed here (only WRWYCRCK inhibits pfTopoI) relative to that of the above mentioned TopoIB type enzymes (inhibited by WYCRCK, KCCRCK, and WRWYCRCK) [40]. To further address the specificity of the pfTopoI active inhibitor WRWYCRCK, we tested the effect of this peptide on the two restriction endonucleases EcoRI and PvuII. Increasing concentrations of the peptide were incubated with the test plasmid (pUC19) before addition of either of the restriction enzymes. As evident from Figure 3, and consistent with previously published results [40], the peptide had no or only very modest effect on the cleavage activity of these enzymes, confirming the specific action of WRWYCRCK.

All three peptides, WYCRCK, KCCRCK, and WRWYCRCK have previously been shown to possess an unspecific DNA binding capacity, which was confirmed in the present study (data not shown) [40]. However, the lack of inhibition of endonuclease activity and the inhibition of pfTopoI activity only by WRWYCRCK and not by WYCRCK and KCCRCK even at very high concentrations argues against peptide inhibition being the result of a simple competition for noncovalent DNA binding. Indeed, for vvTopoI and *λ*-Int, all peptides were demonstrated not to affect noncovalent DNA interaction and it was suggested that inhibition was a result of the peptides preventing the transition from noncovalent to covalent binding, that is, DNA cleavage, by interfering with the enzyme-DNA interphase [40].

### 3.4. Prediction of the Interaction Mode between the Peptide and the pfTopoI-DNA Complex

**Figure 4:**
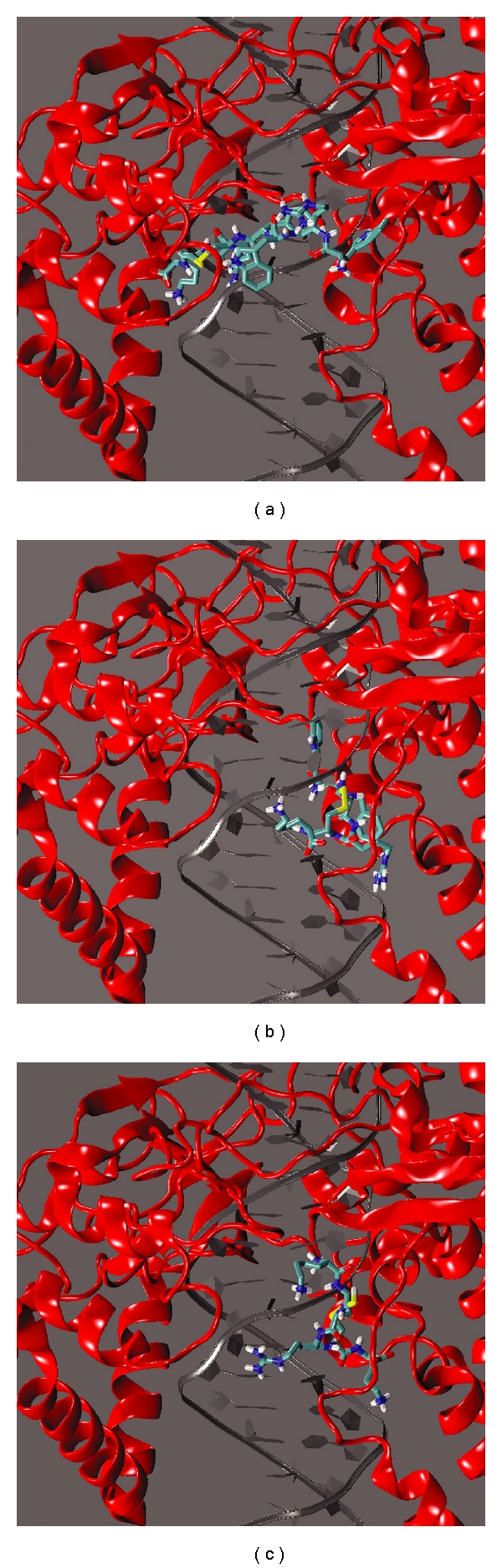
Docking of peptides into the noncovalent pfTopoI-DNA complex. Side view of the ternary pfTopoI-DNA-peptide complex. The structure represents the best complex obtained from the docking experiment. The protein is shown in red cartoon, the DNA in grey ribbon and the peptide in licorice, with the atoms coloured with the following code: carbon: cyan, nitrogen: blue, oxygen: red, sulphur: yellow. (a) Octapeptide WRWYCRCR, (b) hexapeptide WYCRCR, and (c) hexapeptide KCCRCR.

Docking experiments have been carried out to identify the preferential binding site of the WRWYCRCK octapeptide on the noncovalent pfTopoI-DNA complex. 250 docking runs were done and the best complex, having a free energy value of −14.0 Kcal/mol, was selected and analyzed. This complex shows that the peptide is located in the minor groove cavity in front of the active site (see Figure 4(a)), establishing many contacts with both the protein and the DNA bases, as reported in Table 1. Concerning the DNA contacts, interesting interactions occur between the peptide and Gua12-Ade15 and Thy32-Thy34 on the scissile and intact strand, respectively. The optimal positioning of the octapeptide in the minor groove is due either to a good geometrical fitting between the two molecules, or to the high number of electrostatic interactions between the positively charged residues of the peptide and the negatively charged DNA phosphates. As far as the protein is concerned, interesting interactions occur between Trp3 and Cys5 of the peptide and Arg310 of pfTopoI and between Tyr4 and Asp513 of pfTopoI (see Table 1). Residues Arg310 and Asp513 of the *Plasmodium* protein correspond to residues Arg364 and Asp533 of the human enzyme, which are known from the 3D structure of the ternary drug-DNA-enzyme complex to directly interact with the camptothecin drug [55, 56]. The peptide then, positioned in the minor groove of the DNA just in front of the protein active site, exerts an inhibition of the cleavage process thus providing an explanation for the experimental results reported in Figure 2(c). 

An identical docking experiment has been performed also for the two hexapeptides WYCRCK and KCCRCK, not having any inhibitory effect on pfTopoI relaxation. The best docked complexes, having a free energy value of −11.36 and −11.04 Kcal/mol, are reported in Figures 4(b) and 4(c) for the WYCRCK and KCCRCK peptide, respectively. Both peptides are found in a region different from the one found for the octapeptide. The two hexapeptides are located in proximity of the major groove in a region far from the enzyme active site and, in contrast to what was observed for the octapeptide, they are not able to interact with Arg310 and Asp513, providing a structural explanation for their lack of inhibition.

## 4. Discussion

During recent years, bioactive peptides have been suggested as an alternative or complement to traditional small-molecule drugs in the combat against protozoan parasites [10, 24, 55, 57, 58]. One of the suggested advantages of peptide drugs in antiparasite treatment relies on the ease by which such drugs can be selected or modified to target desired biological pathways using nature's own selection mechanisms or large throughput *in vitro *screening and/or directed evolution setups. Another advantage relies on the relatively large interphase between peptide drugs and their target, possibly facilitating an increased specificity of peptide drugs compared to small-molecule drugs [25, 59]. Until recently, high synthesis costs have hampered the possibilities of developing peptide-based drugs against various relevant targets. However, with new synthesis strategies and lowered monomer costs the interest in developing peptide drugs has markedly increased [24–27]. One of the very promising strategies was first presented by Nagaraja's research group, who had taken advantage of antibodies raised by the natural immune response of mice injected with the desired target, in the reported cases, *Mycobacteria* DNA gyrase or TopoI [28–30]. As a result different antibodies with specific inhibitory effects on either target have been identified. Remarkably, these antibodies appear extremely specific and show no activity against the *E. coli *counterparts of the *Mycobacteria* topoisomerases. Hence, these antibodies hold great promise for the further development of *Mycobacteria*-specific peptide drugs based on the amino acid sequence of the active parts of the antibodies [28]. Indeed, several studies highlight the feasibility in deriving active peptides with specificity retained from the antibodies from which they originate [31–34].

Another reported strategy was based on selecting peptides with activity against the TopoI related *λ*-Int from a large library [35]. As a result of this study, a number of peptides with inhibitory effect on the recombinase were identified. Some of these, WYCRCK, KCCRCK, and WRWYCRCK, also inhibited the relaxation activity of vvTopoI [39, 40]. In the present study, we demonstrate that of these peptides, WRWYCRCK but not WYCRCK, or KCCRCK inhibits DNA relaxation mediated by pfTopoI. As previously reported for the peptide inhibition of *λ*-Int and vvTopoI, it is specifically the cleavage reaction of pfTopoI that is inhibited by WRWYCRCK, while ligation is largely unaffected by the peptide, possibly due to the peptide being unable to bind to the covalent pfTopoI-DNA cleavage complexes. The inhibition on cleavage appears to be dependent on cysteine bridging since the addition of DTT counteracts the peptide effect. For *λ*-Int and vvTopoI it was demonstrated that although the peptide does bind DNA unspecifically, the inhibitory effect of active peptides on DNA cleavage could not be ascribed to a simple competition preventing noncovalent DNA interaction of the Topos [40]. Rather the peptides were suggested to prevent the transition from noncovalent to covalent binding. Although this was not addressed experimentally for pfTopoI the inhibition of this enzyme by only one of the peptides, WRWYCRCK, argues for a specific inhibition rather than merely an unspecific competition for DNA binding. Note that all three peptides bind DNA in an unspecific manner [40]. 

This notion is further supported by molecular docking experiments in which the molecular mechanism for the inhibition exerted by the octapeptide was analyzed. This analysis allowed us to predict the preferential interaction interface between the noncovalent pfTopoI-DNA complex and the peptide itself. This is in agreement with the peptide being able to prevent the transition from noncovalent to covalent binding. Hence, the complex with the lowest free energy, that is, the best complex, is represented by the peptide inserted in the DNA minor groove, near the active site (Figure 4), where it impedes the catalytic tyrosine to produce the nick on the scissile strand, as demonstrated by the cleavage assay (Figure 2). Indeed, the peptide interacts with two residues in proximity of the active site, Arg310 and Asp513, which are the plasmodial counterpart for the human residues Arg364 and Asp533 that in the 3D structure of the human enzyme are in direct contact with the camptothecin drug [56]. The peptide is stabilized by numerous contacts to either the protein or the DNA, confirming that it represents an efficient inhibitor of the enzyme. Docking of the two noninhibiting peptides, WYCRCK and KCCRCK, into the noncovalent pfTopoI-DNA complex revealed that these peptides were located far from the active site of pfTopoI, which may explain why they do not inhibit pfTopoI. 

Although, until now, no species-specific peptide inhibitors of parasitic Topos have been reported, we believe that the presented studies demonstrate the feasibility of inhibiting Topos relevant in antiparasite treatment and that molecular docking may pave the road for the rational development of species-specific inhibitors.

## Figures and Tables

**Table 1:** Contacts between the peptide WRWYCRCK, and the noncovalent pfTopoI-DNA complex calculated for the best docked complex.

Peptide	Protein	DNA
Trp1	—	—
Arg2	Lys208, Gly313, Glu314, Ser514	Ade13
Trp3	Tyr205, Lys208, Arg310, Arg312, Gly313	Ade14
Tyr4	Arg312, Gly313, Asp513	Gua12, Ade13, Ade14
Cys5	Tyr205, Arg310, Gly311	Ade14
Arg6	Arg312	Gua12, Ade13, Ade14, Ade15, Thy32, Cyt33
Cys7	Lys322	—
Lys8	—	Ade15, Cyt33, Thy34
